# Release characteristics of Pb and BETX from in situ oil shale transformation on groundwater environment

**DOI:** 10.1038/s41598-021-95509-2

**Published:** 2021-08-09

**Authors:** Han Wang, Wenjing Zhang, Shuwei Qiu, Xiujuan Liang

**Affiliations:** 1 Key Laboratory of Groundwater Resources and Environment (Jilin University), Ministry of Education, Changchun, 130021 China; 2 National-Local Joint Engineering Laboratory of In-Situ Conversion, Drilling and Exploitation Technology for Oil Shale, Changchun, 130021 Jilin China; 3 College of New Energy and Environment, Jilin University, Changchun, 130021 China; 4 School of Water Resources and Environment, Hebei GEO University, Shijiazhuang, 050031 China; 5 Hebei Province Key Laboratory of Sustained Utilization and Development of Water Resources, Shijiazhuang, 050031 China; 6 Hebei Province Collaborative Innovation Center for Sustainable Utilization of Water Resources and Optimization of Industrial Structure, Shijiazhuang, 050031 China

**Keywords:** Environmental chemistry, Environmental impact

## Abstract

Oil shale has received attention as an alternative energy source to petroleum because of its abundant reserves. Exploitation of oil shale can be divided into two types: ex situ and in situ exploitation. In situ transformation has been favoured because of its various advantages. Heating of oil shale leads to the production of oil and gas. To explore the influence of solid residue after pyrolysis of oil shale on the groundwater environment, we performed ultrapure water–rock interaction experiments. The results showed that Pb tended to accumulate in solid residues during pyrolysis. Additionally, the Pb concentration goes up in the immersion solution over time and as the pyrolysis temperature increased. In contrast, when we measured the soaking data of benzene series, the concentrations of benzene and toluene produced at temperatures over 350 ℃ were highest in the four oil shale pyrolysis samples after pyrolysis. The water–rock interaction experiment for 30 days led to benzene and toluene concentrations that were 10^4^ and 1070-fold over the limit of China’s standards for groundwater quality. Over time, the content of benzene series was attenuated via biological actions. The results show that in situ oil shale mining can lead to continuous pollution in the groundwater environment.

## Introduction

Because of the rapidly increasing demand for oil, conventional petroleum resources will eventually be depleted. Thus, unconventional petroleum resources must be developed and utilised^1^. Oil shale is a combustible sedimentary rock containing organic material, fine bedding, and high ash and solid combustible mineral contents and can be considered as an alternative energy source to crude oil^2,3^. The oil content of oil shale is more than 3.5%. Shale oil, dry distillation gas, and semi-coke shale can be obtained by low-temperature dry distillation^4^. The properties of shale oil are very similar to those of petroleum^5^; therefore, existing equipment can be used for oil extraction. The organic matter content generally reaches 40% of the total mass of oil shale^6^, whereas the inorganic matter content (minerals) is higher (generally > 60%). Organic components include organic C, H, O, N, and S. Inorganic components mainly include quartz, muscovite, kaolinite, calcite, and carbonate. In some areas, oil shale contains small amounts of diamond, iron ore, and S, Ti, Cr, Ni, and other elements. The organic matter of oil shale mainly includes kerogen and bitumen^7^. The bitumen content of Chinese oil shale is generally ~ 1%. Organic matter is the main source of shale oil and shale gas during oil shale pyrolysis. Shale oil (calculated based on in situ oil shale) accounts for ~ 400 billion tons of oil worldwide, which is higher than that of traditional crude oil that accounts for > 300 billion tons of oil worldwide^8^.

Oil shale pyrolysis technology can be divided into traditional surface retorting technology and underground in situ transformation technology^9–11^. Oil shale surface retorting leads to several environmental problems such as water–air pollution and waste residue stacking, restricting the large-scale use of oil shale resources^12^. Because of the disadvantages of large-scale investments, high pollution, and high cost of surface retorting technology, various studies of in situ transformation have been conducted worldwide. For example, numerous pilot conversion technologies have been tested on oil shale such as the Shell In Situ Conversion Process, Exxon Mobil Electrofrac technology, and Occidental Modified In Situ technology. In China, technology has been developed by the Zhongcheng Group and Jilin University for in situ conversion experiments on oil shale in the Songliao Basin, which revealed positive results^12^. The technology is mainly used to heat underground oil shale. When the temperature is increased, kerogen in the oil shale releases the shale oil and gas, but also produces large amounts of pollutants^13^. Pollutants remain adsorbed and accumulate in the rock strata for a long period of time; their slow release can cause lasting damage to groundwater and is not conducive to restoration and treatment. Because of the complex composition of pollutants, various pollutants may react chemically with other substances after entering the groundwater or a "synergistic effect" can occur among them, posing a serious threat to the environmental security of groundwater^14–18^.

Few previous studies have focused on the pollution of groundwater caused by in situ oil mining. Hu et al. (2019) reported that in situ oil shale mining aggravates the pollution of groundwater with organic matter and residual oil shale ash^19^. However, the experimental temperature used in the previous study did not reflect the actual oil shale conditions. Numerous studies have focused on the pollution resulting from oil shale heating; when shale is heated to a certain temperature, a series of physical and chemical reactions occurs. As the temperature rises, water begins to evaporate and kerogen decomposes into bitumen, eventually releasing shale oil and gas. With increasing heating temperature, kerogen decomposition increases. During this process, C–H bonds break and recombine to form a series of complex organic compounds^20–22^. Kerogen in oil shale undergoes a variety of reactions during pyrolysis^23^, and the presence of inorganic minerals makes the pyrolysis process of oil shale more complex and diverse^24^. The main organic pollutants in groundwater are benzene series (such as benzene, toluene, ethylbenzene, xylene) and phenolic compounds (phenol, naphthol, aniline, pyridine)^25^.

**Figure 1:**
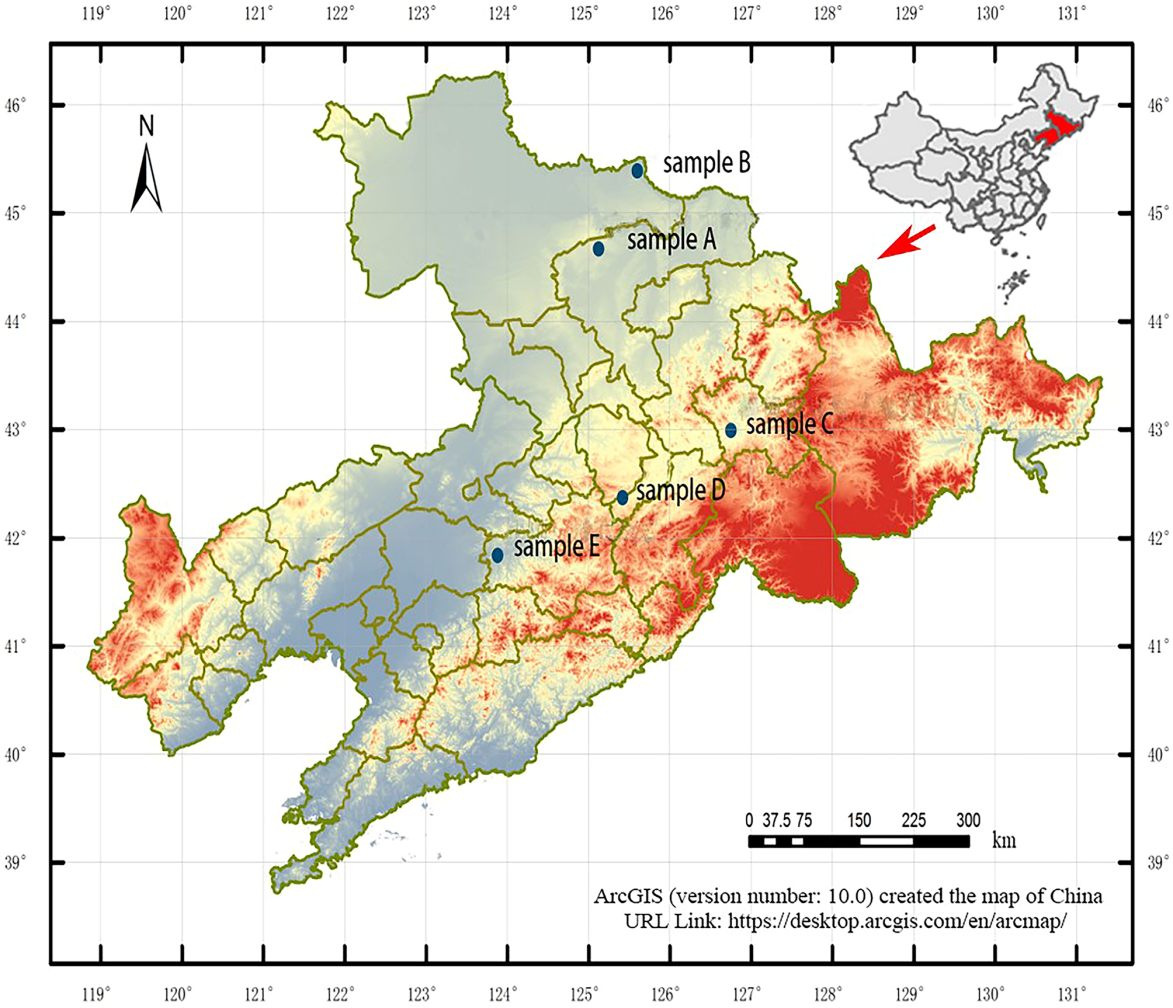
Locations of oil shale sampling points.

In this study, we selected a representative heavy metal, i.e., Pb, to evaluate the release of heavy metals into the groundwater environment. In contrast to organic contaminants, Pb cannot be destroyed but can only be relocated. This element is nonessential to the human body, and excessive intake can damage the nervous, skeletal, circulatory, enzymatic, endocrine, and immune systems in humans^26^. Lead is a contaminant that is generally related to the mining industry. We also evaluated characteristic organic pollutants (benzene, toluene) in the pyrolysis oil–water immersion solution and residue immersion solution by atomic absorption spectrophotometer^25^. X-ray diffraction (XRD) was performed for five samples from oil shale regions (Fig. 1) to determine the effects of clay minerals on Pb release and ion precipitation–dissolution equilibrium in the groundwater.

## Methods

### Sample and water–rock interaction experiment

Five samples (samples A–E) were collected from five oil shale regions in the Jilin and Liaoning Provinces (Fig. 1)^27^. Prior to the water–rock interaction experiments, we placed the samples in a MXG1200-40S tubular furnace and heated them at 200 °C, 350 °C, and 500 °C for 2 h, generating a solid residue. Shale oil can be divided into distillation fractions for use in petroleum refining processes. Different oil components are produced between 300 and 500 °C; oil and gas are mainly produced between 300 and 475 °C^12,28^. Based on the division of oil shale pyrolysis process conditions, we selected 0 °C, 200 °C, 350 °C, and 500 °C as the final temperatures^29^. First, a tube furnace was used to heat oil shale. For the water–rock interaction experiment, 50 g of each of residue was weighed at the same heating temperature in the same area with constant (room) temperature and placed in a 500-mL polyethylene terephthalate bottle with a lid. Ultrapure water was poured into the bottle, and the bottle was closed with the lid. After 0.5, 2, 4, 8, 15, 30, 60, 90, 120, 150, 180, 270, and 360 days, the Pb concentration was measured.

### Determination of lead content

To ensure that the natural sealing and reducing conditions of the groundwater environment were reflected, one bottle was measured only once. The background value of each ion in water was negligible in this experiment. We used atomic absorption to determine the Pb content of the immersion solution.

### Determination of benzene series concentration

To determine the concentration of benzene series (benzene, toluene) present in the immersion solution, liquid chromatography was carried out using a Shimadzu LC-20AT liquid chromatograph (Kyoto, Japan), SPD-20A ultraviolet–visible detector, CTD-10AS temperature storage tank, and ODS column (4.6 mm × 250 cm). The chromatographic conditions were as follows: chromatographic column, ODS column (4.6 mm × 250 mm); mobile phase, 70% methanol; flow rate of mobile phase, 1.0 mL/min; injection volume, 50 µL; column temperature, 40 °C. An appropriate amount of soaking liquid sample was absorbed and filtered through a 0.25-μm filter membrane. Next, 50 μL was absorbed into the injection needle before testing the stable operation of the instrument injection. The concentrations of benzene and toluene were set to 0.1, 1, 2, 5 and 10 mg/L, respectively, using a standard solution of benzene and toluene (concentration of 1 mg/mL) and ultrapure water.

### Clay minerals

XRD can be used to qualitatively describe the compositions of inorganic minerals in oil shale samples before and after the pyrolysis of solid residues^30^. A Shimadzu AA-6000CF atomic absorption spectrophotometer was used to determine the crystal structure parameters, mineral species, and relative kerogen percentage. The samples were dried and crushed to 200-mesh powders by using a sieve. The experimental conditions were as follows: voltage of 40 kV, current of 40 mA, scanning angle (2θ) of the copper electrode (λ = 1.54184) of 5–60°, step length of 0.05, and scanning speed of 1.0°/min.

## Results and discussion

### Clay minerals

**Figure 2:**
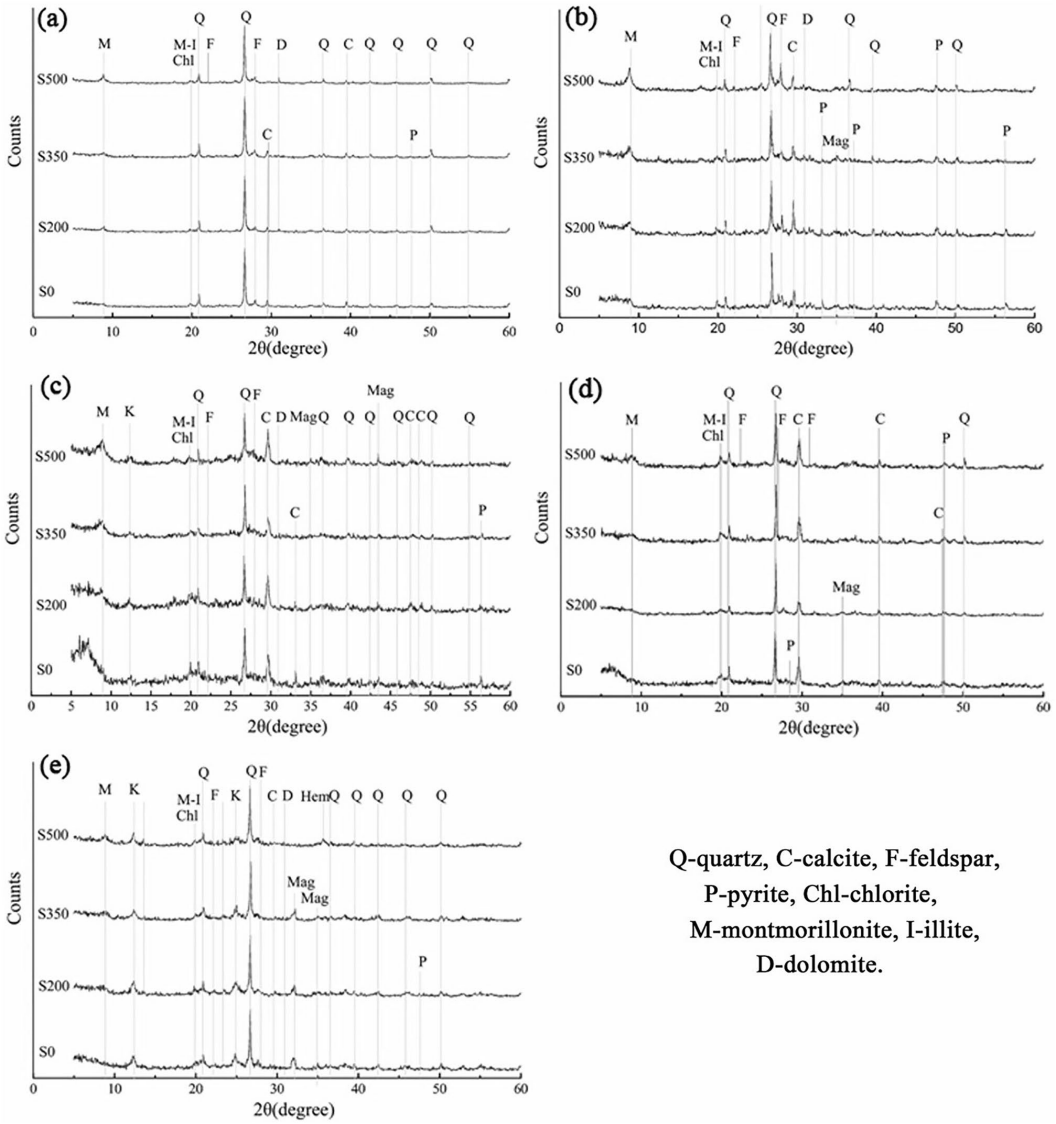
X-ray diffraction data of five samples.

The adsorption of heavy metals by colloidal substances (e.g., clay minerals, inorganic polymers such as water and oxides, and organic polymers such as humus) in natural water affects the conversion of heavy metals in the water environment. In nature, the heavy metal concentration of the aqueous phase is very low. The main enrichment and solid phases are related to colloid adsorption. Oil shale contains large amounts of clay minerals, such as montmorillonite, illite, and kaolinite, which strongly adsorb heavy metals. This characteristic also provides the main pathway of heavy metal transfer from an unsaturated solution to the solid phase. To analyse the mineral characteristics, XRD data (Fig. 2) were automatically extracted and smoothened. According to the XRD patterns, clay minerals (Aimon mixers) were the main inorganic minerals present, followed by quartz and carbonate minerals and a small amount of pyrite. The mineralogical composition of the five samples is shown in Table 1.

**Table 1:** Mineralogical composition of five samples based on X-ray diffraction (XRD) analysis.

Sample	Mineral composition and content (%)						
	Quartz	Feldspar	Calcspar	Chlorite	Kaolinite	White mica	I/S
A	12	8	7	5	–	–	68
A 200 ℃	13	7	5	2	–	10	63
A 350 ℃	11	7	6	6	–	7	63
A 500 ℃	13	8	1	6	–	15	58
B	5	5	8	10	–	5	67
B 200 ℃	4	9	12	4	–	8	67
B 350 ℃	4	6	6	3	–	10	71
B 500 ℃	5	12	8	2	–	13	60
C	3	–	8	8	–	4	77
C 200 ℃	3	–	8	8	–	4	77
C 350 ℃	4	3	9	4	–	8	72
C 500 ℃	4	3	13	4	–	18	68
D	5	4	–	2	10	–	79
D 200 ℃	6	3	1	3	12	–	75
D 350 ℃	5	1	1	3	7	1	82
D 500 ℃	6	2	–	2	8	8	74
E	4	–	12	6	–	–	78
E 200 ℃	8	5	10	6	–	–	71
E 350 ℃	5	1	11	5	–	–	78
E 500 ℃	4	2	12	4	–	–	78

*I/S* illite/montmorillonite mix.

As shown in Table 1 and Fig. 2, all samples mainly contained montmorillonite, illite/montmorillonite, quartz, feldspar, dolomite, calcite, and chlorite. Based on the comparison of the XRD spectra of heated and unheated samples, the peak height of montmorillonite increased with increasing temperature, indicating that dehydration of clay minerals had occurred. When those samples were placed in water, clay minerals recombined with the water and released the adsorbed Pb.

### Lead content

**Figure 3:**
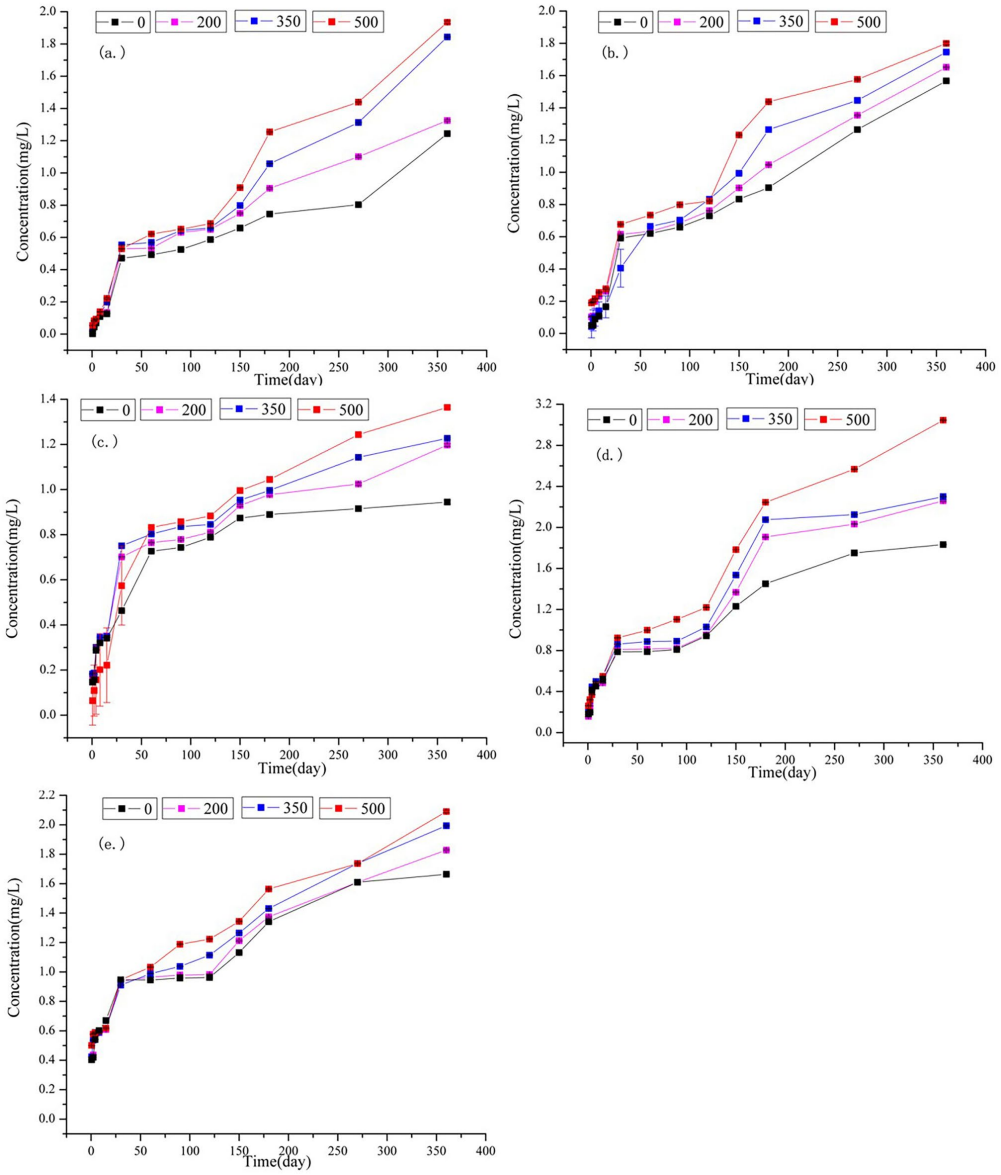
Change in Pb concentration over time in the five samples.

Figure 3 shows the change in the Pb concentration over time for unheated (0 °C) and heated (200 °C, 350 °C, 500 °C) samples. The Pb concentration gradually increased over time. The concentrations of Pb ions in the residues of the immersion solution of all five samples after pyrolysis were higher than that in the original rock^31^. Monitoring of the pH during this experiment showed that the pH range was 7–10. As Pb exists in various forms of Pb^2+^ hydroxide in aqueous solution, the pH also affects the amount of Pb in aqueous solution. The amount of Pb in aqueous solution increases under acidic or alkaline conditions.

Pb concentrations were high in residues and Pb was easily soluble in water. The overall change in the Pb concentration was consistent; i.e., it gradually increased over time and with increasing pyrolysis temperatures^32,33^. With increases in the pyrolysis temperature, heavy metals are incorporated into the solid residue. The presence of heavy metals in oil shale is important factor in its leaching behaviour. Studies have shown that heavy metal elements in oil shale exist in various states^34,35^: water-soluble state, ion-exchange state, carbonate-binding state, sulphide-binding state, organic-binding state, and residue state. In general, heavy metal elements in water-soluble, ion-exchange, and carbonate-binding states can be easily dissolved in the environment. The sum of these three states is the potential eluvial state of the element, and the value of the set of three states determine the migration degree of the element. Thus, heavy metals in water can be hydrolysed to form hydroxides, sulphides, or carbonates with their corresponding anions, and a precipitation–dissolution equilibrium is eventually reached.

The adsorption of heavy metals by colloidal substances (clay minerals, inorganic polymers such as water and oxides, organic polymers such as humus) in natural water has an important effect on the conversion of heavy metals in the groundwater environment. Adsorption is the main means by which heavy metals are transferred from an unsaturated solution to the solid phase. In the natural state, the amount of heavy metals is very low in the aqueous phase, whereas the main enrichment and solid phase are largely related to colloid adsorption. Clay minerals are the most important and complex components of inorganic colloids in the environment. They are formed during the weathering process of primary minerals and consist of aluminosilicates. Oil shale contains numerous clay minerals such as montmorillonite, illite, and kaolinite, which strongly adsorb heavy metals.

In water, lead mainly exists as Pb^2+^, and its content and form are clearly affected by CO_3_^2−^, OH^−^, and Cl^−^, etc. Lead can exist in various forms, such as PbOH^+^, Pb(OH)_2_, PB(OH)_3_^−^, PbCl^+^, and PbCl_2_.

The major dissolution and complexation equilibria of lead compounds are as follows^36^.

- Solubility equilibrium:
  $$\begin{aligned} & {\text{PbCO}}_{3} = {\text{Pb}}^{2 + } + {\text{CO}}_{3}^{2 - } \\ & {\text{Pb}}\left( {{\text{OH}}} \right)_{2} = {\text{Pb}}^{2 + } + 2{\text{OH}}^{ - } \\ & {\text{PbSO}}_{3} = {\text{Pb}}^{2 + } + {\text{SO}}_{4}^{2 - } \\ & {\text{Pb}}_{3} \left( {{\text{OH}}} \right)_{2} \cdot \left( {{\text{CO}}_{3} } \right)_{2} = 3{\text{Pb}}^{2 + } + 2{\text{OH}}^{ - } + 2{\text{CO}}_{3}^{2 - } \\ \end{aligned}$$
- Complex balance:
  $$\begin{aligned} & {\text{Pb}}^{2 + } + {\text{OH}}^{ - } = {\text{Pb}}\left( {{\text{OH}}} \right)^{ + } \\ & {\text{Pb}}^{2 + } + 2{\text{OH}}^{ - } = {\text{Pb}}\left( {{\text{OH}}} \right)_{2} \\ & {\text{Pb}}^{2 + } + 3{\text{OH}}^{ - } = {\text{Pb}}\left( {{\text{OH}}} \right)_{3}^{ - } \\ & {\text{Pb}}^{2 + } + {\text{Cl}}^{ - } = {\text{PbCl}}^{ + } \\ & {\text{Pb}}^{2 + } + 2{\text{Cl}}^{ - } = {\text{PbCl}}_{2} \\ \end{aligned}$$

### Benzene series content

**Figure 4:**
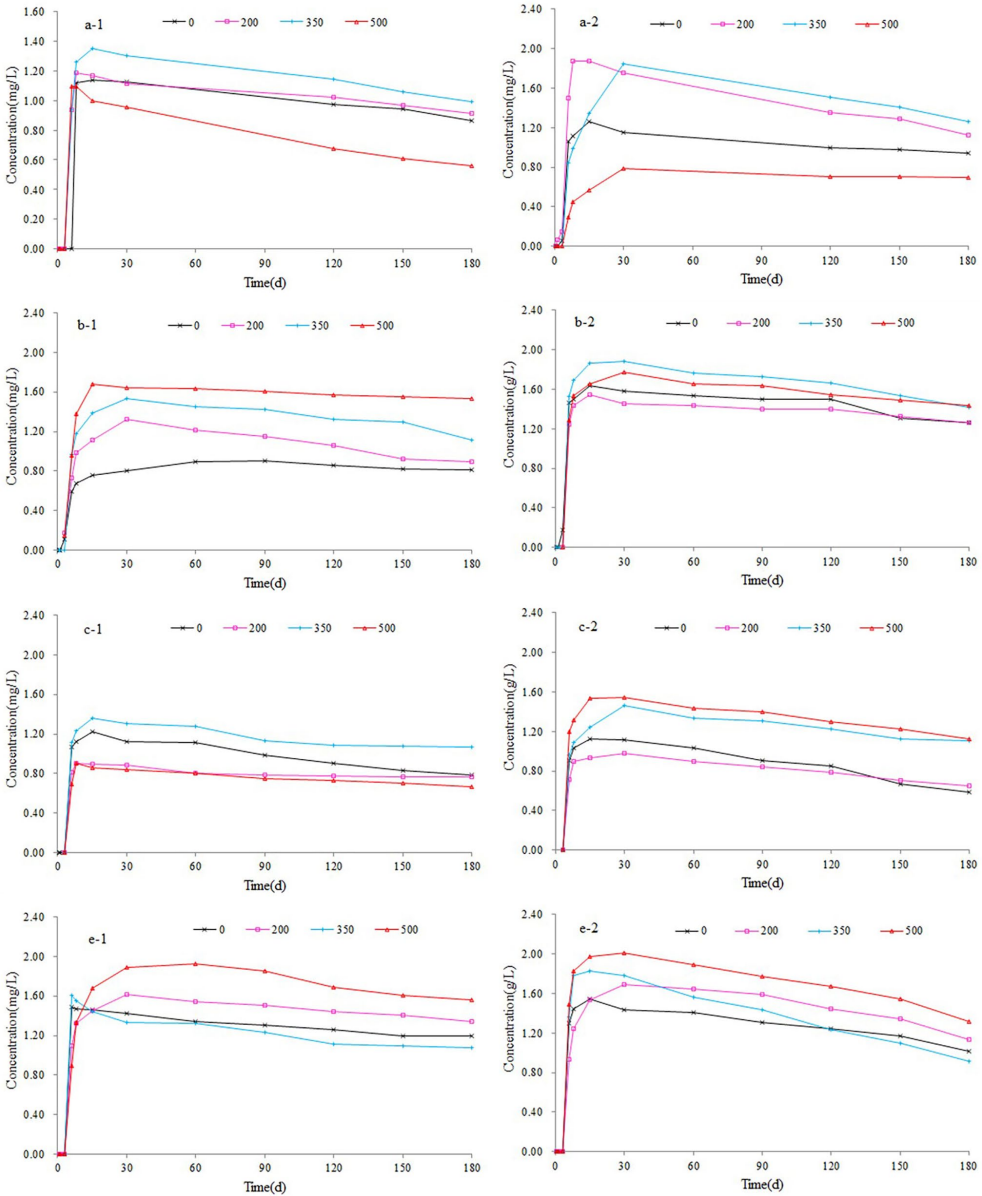
Change in the benzene series concentration over time in the four samples, where -1 indicates benzene and -2 indicates toluene.

Figure 4 shows that the concentration of benzene and toluene in each region first increased and then decreased over time, showing the same overall trend. The benzene series was not detected in the water of sample A for the first 5 days and began to appear on approximately day 6, reaching a peak of more than 1 mg/L on day 15. At 350 °C, the peak value of benzene series was higher than 1.3 mg/L. Over days 15–180, the concentration only slightly changed and decreased, indicating that the benzene series in water began to decay naturally. The final concentration of benzene series in water was less than the initial concentration. The benzene series in sample B began to appear in the water at around 3 days, reaching a peak of more than 0.9 mg/L on day 30. At 500 °C, the peak value of benzene series was above 1.7 mg/L. Over days 30–60, the concentration slightly changed and then decreased, indicating that the benzene series in water began to decay naturally. The final concentration of benzene series in water was less than the initial concentration. The benzene series in sample C began to appear in the water at around 6 days, reaching a peak of more than 0.9 mg/L on day 15. At 350 °C, the peak value of benzene series was above 1.4 mg/L. Over days 15–180, the concentration slightly changed and then decreased, indicating that the benzene series in water began to decay naturally. The final concentration of the benzene series in water was less than the initial concentration. The benzene series in sample E began to appear in the water at around 6 days, reaching a peak of more than 1.5 mg/L on day 15. At 500 °C, the peak value of the benzene series was above 1.9 mg/L. Over days 30–180, the concentration slightly changed and then decreased, indicating that the benzene series in water began to decay naturally. The final concentration of the benzene series in water was less than the initial concentration. The initial concentration of the benzene series was related to kerogen content in oil shale.

The results showed that the benzene series underwent natural attenuation in the aquifer, which occurred through a series of complex mechanisms such as dilution, volatiles, convection, dispersion, adsorption, and analysis and involved microorganisms^37,38^. As the concentration of each contaminated component decreased, the diffusion velocity also decreased. The contribution of various mechanisms to the natural decay of benzene series varies. Important roles include the dilution effect, volatilization, adsorption analysis, and microbial degradation.

In this study, the dilution effect and volatilization were not considered because the experiment was performed in a polyethylene terephthalate bottle filled with water. Adsorption is an important factor in the attenuation of benzene series in aquifers as nonpolar organics, such as benzene, and are easily adsorbed by organic matter on the mineral surface^39^. The adsorbed amount of organic matter in the mineral medium depends on the content of organic carbon, and the adsorption amount increases with increasing organic carbon contents^40^. After soaking for a long period, colony formation and microbial action were observed in the soaking bottle^41–43^. Thus, biodegradation is an important environmental process in the decomposition of benzene series.

As shown in Fig. 4, benzene and toluene first increased over time, and then decreased after reaching their maximum values. The natural decay of benzene series conformed to first-order reaction kinetics with the following equation:

$${\text{C}} = {\text{C}}_{0} \cdot {\text{e}}^{{ - \lambda {\text{t}}}}$$

where C is the concentration of material removed by natural attenuation at time t; C_0_ is the initial concentration, λ is the material attenuation coefficient, and the unit is the inverse of time. A larger λ indicates a faster decay rate.

**Figure 5:**
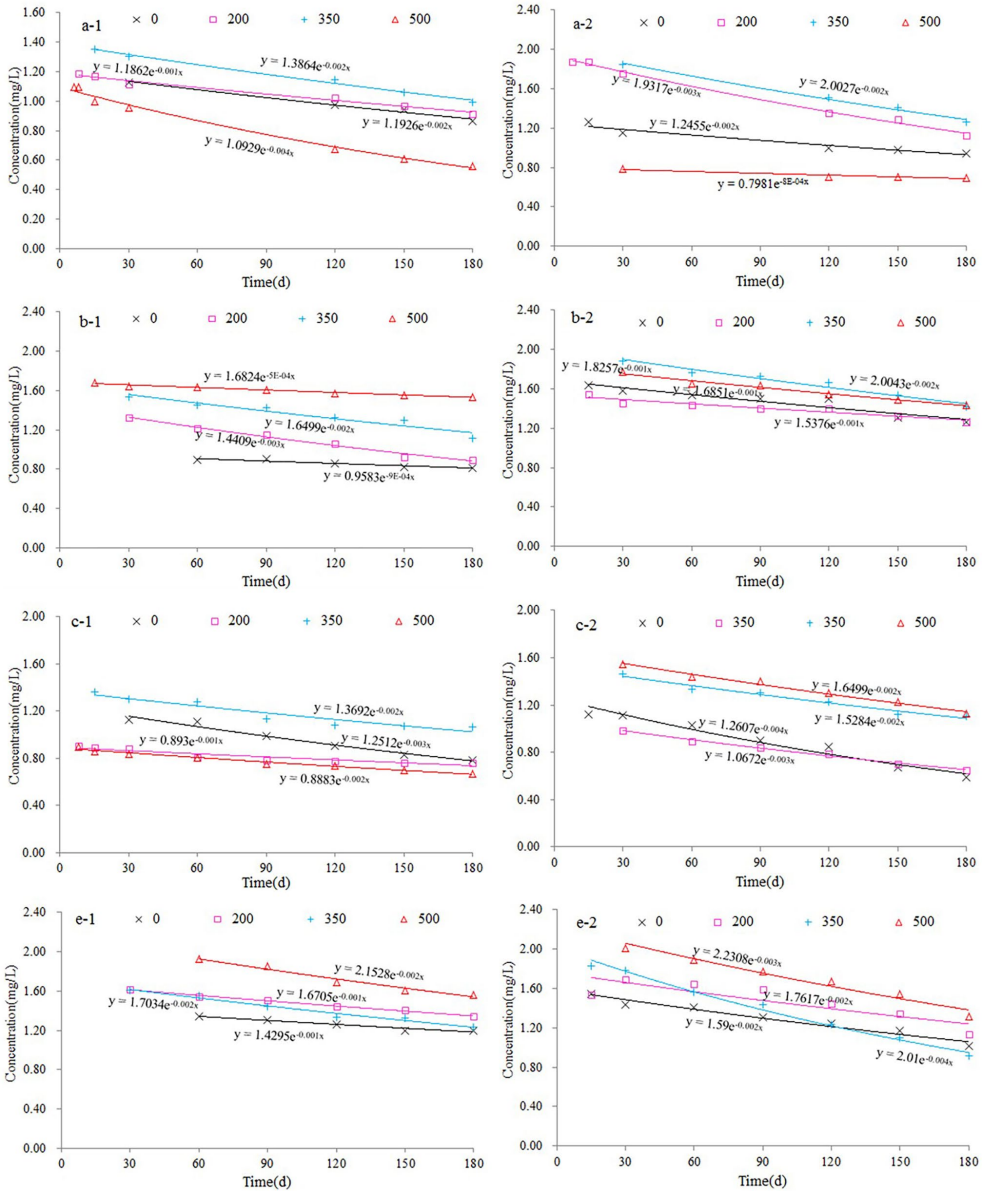
First-order decay kinetic equation of benzene and toluene.

Figure 5 shows the first-order kinetic equation of benzene and toluene in the natural decay process and that of benzene and toluene in the natural decay of crude oil shale rocks and residues following pyrolysis at different temperatures. Table 2 shows the determination coefficients (R2) for Fig. 5.

**Table 2:** The determination coefficients (R^2^) for the regression of samples.

	R^2^-Benzene	R^2^-Toluene
A-0	0.9789	0.9489
A-200	0.983	0.9936
A-350	0.9903	0.9849
A-500	0.991	0.8959
B-0	0.8885	0.897
B-200	0.9775	0.9232
B-350	0.9153	0.9589
B-500	0.9743	0.9819
C-0	0.9759	0.96
C-200	0.8525	0.993
C-350	0.9011	0.9709
C-500	0.9805	0.9841
E-0	0.9537	0.9603
E-200	0.9932	0.7485
E-350	0.9722	0.9892
E-500	0.963	0.9556

## Conclusion

In this study, clay minerals (Aemon mixers) were the predominant inorganic minerals in all five samples, followed by quartz and carbonate minerals and a small amount of pyrite. The pyrolysis temperature affected the composition of solid oil shale residue. The XRD data showed that the clay minerals lost water with increasing pyrolysis temperature. The overall trend of the heavy metal ion Pb was consistent, increasing gradually over time and with increasing pyrolysis temperatures. This is because the weight loss of oil shale differs at different temperatures, leading to an increased number of heavy metal ions per unit weight. Based on the long-term water–rock interaction, the adsorption of clay minerals reached a dissolution–complexation equilibrium. After 360 days of water–rock interaction (Fig. 3), the rate of increase in the heavy metal concentration slowed, and the concentration of each sample was > 1.4 mg/L (one sample reached 3.0 mg/L). According to the General Administration of Quality Supervision, Inspection & Quarantine of China Standards for groundwater quality (GB/T 14848-2017), in situ transformation will result in a Pb content of over tenfold the standard value. Semi-coke present after oil shale in situ transformation will lead to continuous heavy metal pollution of the groundwater environment. The contents of heavy metal elements in solution are affected by the dissolution equilibrium. The concentrations of benzene and toluene in each sample increased first and then decreased over time, showing the same overall trend. These concentrations at 350 °C and 500 °C were highest in the four regions after pyrolysis. The benzene concentration of each sample was > 1 g/L (one sample reached 1.9 g/L), whereas the toluene concentration of each sample was > 1.5 g/L (one sample reaches 2.0 g/L). According to the General Administration of Quality Supervision, Inspection & Quarantine of China Standards (GB/T 14848-2017), in situ transformation will result in concentrations of benzene and toluene that are 10^4^- and 1070-fold over the standard limit. The concentration of benzene series was affected by adsorption and microbial degradation, and the attenuation rate of benzene was lower than that of toluene. Oil shale is currently being tested in the field, and a solution for heavy metal pollution must be developed.
